# Establishment of an Efficient Protoplast Regeneration and Transfection Protocol for Field Cress (*Lepidium campestre*)

**DOI:** 10.3389/fgeed.2021.757540

**Published:** 2021-11-16

**Authors:** Sjur Sandgrind, Xueyuan Li, Emelie Ivarson, Annelie Ahlman, Li-Hua Zhu

**Affiliations:** Department of Plant Breeding, Swedish University of Agricultural Sciences, Lomma, Sweden

**Keywords:** Braccicaceae, CRISPR/Cas9, oilseed crop, domestication, protoplast regeneration, transfection

## Abstract

Field cress (*Lepidium campestre*) is a potential oilseed crop that has been under domestication in recent decades. CRISPR/Cas9 is a powerful tool for rapid trait improvement and gene characterization and for generating transgene-free mutants using protoplast transfection system. However, protoplast regeneration remains challenging for many plant species. Here we report an efficient protoplast regeneration and transfection protocol for field cress. Important factors such as type of basal media, type/combination of plant growth regulators, and culture duration on different media were optimized. Among the basal media tested, Nitsch was the best for protoplast growth in MI and MII media. For cell wall formation during the early stage of protoplast growth, relatively high auxin concentrations (0.5 mg L^−1^ NAA and 2,4-D), without addition of cytokinin was preferred for maintaining protoplast viability. After cell wall formation, 1.1 mg L^−1^ TDZ combined with either 0.05 mg L^−1^ NAA or 2,4-D was found to efficiently promote protoplast growth. On solid shoot induction medium, 1.1 mg L^−1^ TDZ without any auxin resulted in over 80% shoot generation frequency. A longer culture duration in MI medium would inhibit protoplast growth, while a longer culture duration in MII medium significantly delayed shoot formation. Using this optimized protoplast regeneration protocol, we have established an efficient PEG-mediated transfection protocol using a vector harboring the *GFP* gene, with transfection efficiencies of 50–80%. This efficient protoplast protocol would facilitate further genetic improvement of field cress via genome editing, and be beneficial to development of protoplast regeneration protocols for related plant species.

## Introduction

Domestication of new plant species has the potential to increase food security by increasing crop diversity and utilize marginal arable land. It is however a tedious and slow process using traditional breeding methods for domestication, as wild species typically carry many undesirable traits for agriculture. Molecular mechanisms underlying important domestication-related traits such as pod shattering, yield, flowering time, seed dormancy etc. have been identified in both model and crop species (Doebley et al., 2006). As such, employing modern breeding technologies holds great potential for speeding up the domestication process of wild species. The latest gene editing technique CRISPR/Cas9 is an efficient and powerful tool for functional analysis of important genes, and can be used to drastically increase the domestication speed. This technique has been used successfully in domestication efforts of plant species such as pennycress (McGinn et al., 2019), wild tomato (Li et al., 2018; Zsögön et al., 2018), and groundcherry (Lemmon et al., 2018).

The delivery of CRISPR/Cas9 vectors is commonly performed by *Agrobacterium*-mediated stable transformation, resulting in transgenic plants that are strictly regulated in some countries. Furthermore, the integration of CRISPR/Cas9 DNA into the plant genomes can cause insertional gene disruption, and increase the likelihood of off-target mutations due to constant expression of the CRISPR/Cas9 system (Zhang et al., 2019). Polyethylene glycol (PEG)-mediated transfection is an alternative and effective approach to deliver CRISPR/Cas9 vectors or ribonucleoprotein complexes into protoplasts, which enables generation of transgene-free mutated lines (Woo et al., 2015). The CRISPR/Cas9 protoplast transfection system has been used successfully to edit genes in several plant species (Kim et al., 2017; Liang et al., 2017; Lin et al., 2018; González et al., 2020; Li et al., 2021), and the protoplast transfection system has also been successfully used for gene editing in plants by base editors (Molla et al., 2020) or prime editing (Lin et al., 2020). However, as protoplast regeneration remains a major obstacle for obtaining mutated lines for most plant species, the method has mainly been used to evaluate mutation efficiencies of sgRNAs of target genes, not for trait improvement in general. An efficient and reliable protoplast regeneration method is thus a prerequisite for crop improvement by CRISPR/Cas9 using the protoplast system.

Field cress (*Lepidium campestre*) belongs to the Brassicaceae family and has a great potential to become a new crop for plant oil production. It is very cold-hardy and can thus be grown in regions where other winter oilseed crops cannot be cultivated, greatly expanding the possible planting region for oilseed crops. Furthermore, it has a high yield potential and some good agronomic traits such as an upright stature, synchronous seed maturity, and resistance to the pollen beetle (Merker and Nilsson, 1995; Bertholdsson, 2017). Due to its biennial nature, field cress has also shown its potential as a catch crop with a positive effect on the yield of barley when it was undersown (Merker et al., 2010). This cropping system could reduce nutrient leaching and tillage, providing valuable ecosystem services and reducing on-farm energy-consumption. Field cress has been under domestication in the last few decades, and has been improved via genetic transformation (Ivarson et al., 2013, 2016, 2017a, b) and marker assisted breeding (Gustafsson et al., 2018; Geleta et al., 2020). Development of an efficient protoplast regeneration and transfection method could facilitate the use of CRISPR/Cas9 for rapid trait improvement, and thus further speed up the domestication process of the species.

In this study, we have studied some important factors affecting protoplast regeneration and transfection, and have successfully established an efficient protocol for protoplast regeneration and transfection of field cress. This method is now routinely used in our lab for trait improvement of the species through genome editing by CRISPR/Cas9.

## Materials and Methods

### Plant Material and *in vitro* Growth Conditions

Seeds from field cress (*L. campestre* L.), accession no. 94–7, were used in this study. This accession was initially collected in Öland, Sweden, and further multiplied in greenhouse. All *in vitro* cultures were maintained in a climate chamber with a temperature of 23 °C/18 °C (day/night) and 16 h photoperiod with a light intensity of 40 μmol m^−2^ s^−1^ (cool white fluorescent tubes).

### Seed Germination

Seeds were surface sterilized in 15% (v/v) calcium hypochlorite (Ca(ClO)_2_) for 20 min, followed by rinsing with sterile water. Surface sterilized seeds were planted on germination medium (half strength MS, 10 g L^−1^ sucrose, 7 g L^−1^ Bacto agar, pH 5.7) in sterile plastic containers, which were placed in the climate chamber as stated above.

### Protoplast Isolation and Culture

Protoplast isolation was based on the Arabidopsis protocol developed by Yoo et al. (2007), with some modifications. About 40–50 fully opened true leaves of 3–4 week old field cress seedlings were finely sliced and incubated in plasmolysis solution (0.4 M mannitol, pH 5.7) for 30 min in the dark at room temperature (RT). After removing the plasmolysis solution, 10 ml enzyme solution (1.5% (w/v) cellulase Onozuka R-10 (Yakult Pharmaceutical Co., LTD, Tokyo, Japan), 0.6% (w/v) Macerozyme R-10 (Yakult Pharmaceutical Co., LTD.), 0.4 M mannitol, 10 mM MES, 0.1% (w/v) BSA, 1 mM CaCl_2_, 1 mM β-mercaptoethanol, pH 5.7) were added and incubated in the dark at RT for 14–16 h with gentle shaking.

The enzyme solution was then diluted with 30 ml W5 solution without glucose (Menczel et al., 1981), filtered through a 40 µm nylon cell strainer, and the protoplasts were collected by centrifugation at 100 *g* for 10 min. After removing the supernatant, the pellet was gently resuspended in 10 ml W5, and centrifuged at 100 *g* for 5 min using a swing-bucket rotor. This washing step was repeated once. Afterwards, the pellet was resuspended in 5 ml W5 and incubated on ice for 30 min in the dark to allow intact protoplasts to sink naturally. The supernatant was removed and the protoplasts were resuspended in 10 ml W5. A sample of the protoplast solution was loaded on a Hemocytometer and observed under a light microscope at ×20 magnification to estimate the amount of intact viable protoplasts isolated. The solution was then centrifuged for 3 min at 100 *g*. After removing the supernatant, the protoplast density was adjusted to 0.4 to 0.6 million protoplasts per ml with 0.5 M mannitol. Sodium alginate solution (2.6% (w/v) sodium alginate, 0.4 M mannitol) was added to the protoplast solution in a 1:1 ratio, and, after gentle mixing, 500 µL aliquots of the suspension were pipetted onto calcium-agar plates (0.4 M mannitol, 2.2 g L^−1^ CaCl_2_, 10 g L^−1^ Phyto agar) for making alginate disks and incubated at RT for 30 min. Thereafter, approximately 2 ml calcium-solution (50 mM CaCl_2_, 0.4 M mannitol) was added onto each disk, and incubated for 1 h at RT to complete the polymerization. The disks were finally transferred to MI medium, which consisted of different basal media and PGRs (Tables 1, 2), in 6-well sterile tissue culture plates. The plates were covered with aluminum foil and transferred to the climate chamber with conditions as stated above. After 24 h, the foil was replaced with fiber cloth to provide a dim lighting for ensuring callus formation. After 3–20 days, the MI medium was replaced with MII medium, which consisted of different PGRs (Table 3). The MII medium was renewed every 5–7 days until protoplast colonies reached a size of approximately 0.1–0.2 mm in diameter.

**TABLE 1:** Effect of basal medium on protoplast viability of field cress.

Basal medium	Protoplast viability in MI medium (%)^a^	Protoplast viability in MII medium (%)^a^	Regeneration (%)^b^
MS	0.0 c	0.0 c	0.0 b
½ MS	0.0 c	0.0 c	0.0 b
Kao	49.7 b	0.0 c	0.0 b
B5	49.7 b	10.0 b	0.0 b
Nitsch	80.0 a	80.0 a	75.0 a

MI medium composition: Basal medium, 10 g L^−1^ sucrose, 10 g L^−1^ glucose, 100 g L^−1^ mannitol, 100 mg L^−1^ casein, 0.5 mg L^−1^ 2,4-D, 0.5 mg L^−1^ NAA, pH 5.7. MII medium composition: Basal medium, 10 g L^−1^ sucrose, 10 g L^−1^ glucose, 100 g L^−1^ mannitol, 100 mg L^−1^ casein, 1.1 mg L^−1^ TDZ, 0.05 mg L^−1^ 2,4-D, pH 5.7. SIM medium composition; MS, sucrose 15 g L^−1^, 1.1 mg L^−1^ TDZ, 0.5 mg L^−1^ AgNO_3_, 2.5 g L^−1^ Gelrite, pH 5.7.

a Protoplast viability was indicated by being round, compact in form, and green in color, observed under light microscope. The results were recorded after 7 days in MI, and 14 days in MII.

b The results were recorded after 4 months. Values followed by the same letter were not statistically different at *p*=0.05 (n = 3).

**TABLE 2:** Effect of PGRs in MI medium on protoplast viability of field cress.

PGR in conc. (mg L^−1^)	Protoplast viability (%)^a^	PGR in conc. (mg L^−1^)	Protoplast viability (%)^a^
TDZ 1.1 2,4-D 1.0	0.0 c	BAP 0.5 NAA 0.5 2,4-D 0.5	19.7 b
TDZ 1.1 2,4-D 0.5	0.0 c	NAA 0.5 2,4-D 0.5	80.0 a
TDZ 1.1 2,4-D 0.25	0.0 c	BAP 2.0 NAA 0.5	0.0 c

MI medium composition: 2.18 g L^−1^ Nitsch, PGRs, 10 g L^−1^ sucrose, 10 g L^−1^ glucose, 100 g L^−1^ mannitol, 100 mg L^−1^ casein, pH 5.7.

a Protoplast viability was indicated by being round, compact in form, and green in color, observed under light microscope after 7 days. Values followed by the same letter were not statistically different at *p*=0.05 (n = 3).

**TABLE 3:** Effect of PGRs in MII medium on protoplast callus formation of field cress.

PGR in conc. (mg L^−1^)	Callus formation (%)^a^	PGR in conc. (mg L^−1^)	Callus formation (%)^a^
BAP 1.0 Zeatin 0.6 NAA 0.5	0.0 b	TDZ 1.1 NAA 0.1	0.0 b
BAP 1.0 Zeatin 0.6 NAA 0.1	0.0 b	TDZ 1.1 NAA 0.05	75.7 a
TDZ 1.1 Zeatin 0.6 NAA 0.1	0.0 b	TDZ 1.1 2,4-D 0.1	0.0 b
TDZ 2.2 NAA 0.1	0.0 b	TDZ 1.1 2,4-D 0.05	80.0 a

MII medium composition: 2.18 g L^−1^ Nitsch, PGRs, 10 g L^−1^ sucrose, 10 g L^−1^ glucose, 100 g L^−1^ mannitol, 100 mg L^−1^ casein, pH 5.7.

a Protoplast colonies formed with a size of ≥0.1 mm in diameter after 30 days. Values followed by the same letter were not statistically different at *p*=0.05 (n = 3).

### Protoplast Regeneration

Microcalli from the alginate disks were spread directly onto shoot induction medium (SIM) (Tables 4–6). The microcalli were subcultured to fresh medium every 3–4 weeks until shoots had appeared. Shoots were transferred to shoot elongation medium (SEM) (MS, 20 g L^−1^ sucrose, 0.05 mg L^−1^ 6-benzyladenine (BAP), 0.03 mg L^−1^ gibberellic acid (GA_3_), 7 g L^−1^ Bacto agar, pH 5.7).

**TABLE 4:** Effect of PGRs in SIM medium on shoot regeneration of field cress.

PGR in conc. (mg L^−1^)	Appearance of calli by visual observation	Regeneration (%)^a^
TDZ 1.1	Normal and green	82.0 a
TDZ 1.1, NAA0.1	Big and hard	0.0 c
TDZ 1.1, NAA 0.01, GA_3_ 0.1	Small, yellow, and hard	5.0 b
TDZ 2.2, NAA0.1	Big and hard	0.0 c
Zeatin 2.0, NAA0.1	Yellow and hard	0.0 c
Zeatin 1.0, NAA0.1	Yellow and hard	0.0 c
Zeatin 2.0, NAA 0.01, GA_3_ 0.1	Small, yellow, and hard	0.0 c
BAP 2.0, NAA 0.1	Small and yellow	1.0 c

SIM medium composition: MS, PGRs, 15 g L^−1^ sucrose, 0.5 mg L^−1^ AgNO_3_, 2.5 g L^−1^ Gelrite, pH 5.7.

a The results were recorded after 4 months. Values followed by the same letter were not statistically different at *p*=0.05 (n = 3).

**TABLE 5:** Effect of C-source in SIM medium on shoot regeneration of field cress.

C-source in conc. (g L^−1^)	Regeneration (%)^a^
Sucrose 15	80.0 a
Sucrose 30	46.7 c
Glucose 10	67.0 b
Glucose 20	45.0 c

SIM medium composition; MS, sugar, 1.1 mg L^−1^ TDZ, 0.5 mg L^−1^ AgNO_3_, 2.5 g L^−1^ Gelrite, pH 5.7.

a The results were recorded after 4 months. Values followed by the same letter were not statistically different at *p*=0.05 (n = 3).

**TABLE 6:** Effect of cytokinin in SIM medium on shoot regeneration of field cress.

Cytokinin conc. (mg L^−1^)	Regeneration (%)^a^
TDZ 0.5	13.3 c
TDZ 1.1	88.8 a
TDZ 2.2	40.0 b
BAP 2.0	0.0 c

SIM Medium composition: MS, PGR, 15 g L^−1^ sucrose, 0.5 mg L^−1^ AgNO_3_, 2.5 g L^−1^ Gelrite, pH 5.7.

a The results were recorded after 4 months. Values followed by the same letter were not statistically different at *p*=0.05 (n = 3).

For optimizing protoplast regeneration efficiency, various MI, MII, and SIM medium compositions and culture durations in MI and MII medium (Table 7 in the result section) were tested.

**TABLE 7:** Effect of culture duration in MI or MII medium on shoot regeneration of field cress.

Medium	Regeneration (%)^a^							
	3 days	5 days	10 days	15 days	20 days	30 days	40 days	50 days
MI^b^	53.3 a	50.0 b	9.0 c	0.0 days	0.0 days	**-**	**-**	**-**
MII^c^	**-**	**-**	0.0 e	9.7 days	40.0 b	55.0 a	19.3 c	5.0 de

MI medium composition: 2.18 g L^−1^ Nitsch, 10 g L^−1^ sucrose, 10 g L^−1^ glucose, 100 g L^−1^ mannitol, 100 mg L^−1^ casein, 2.2 mg L^−1^ NAA, 0.5 mg L^−1^ 2,4-D, pH 5.7. MII medium composition: 2.18 g L^−1^ Nitsch, 10 g L^−1^ sucrose, 10 g L^−1^ glucose, 100 g L^−1^ mannitol, 100 mg L^−1^ casein, 1.1 mg L^−1^ TDZ, 0.05 mg L^−1^ 2,4-D, pH 5.7. SIM medium composition; MS, 15 g L^−1^ sucrose, 1.1 mg L^−1^ TDZ, 0.5 mg L^−1^ AgNO_3_, 2.5 g L^−1^ Gelrite, pH 5.7.

a The results were recorded after 2 months. Values followed by the same letter were not statistically different at *p*=0.05 (n = 3).

b Protoplasts were cultured 25 days in MII medium prior to transfer to SIM medium.

c Protoplasts were cultured 3–5 days in MI.

The detailed information about the medium compositions is given in Tables 1–7.

### Statistical Analysis

For evaluating the protoplast viability, protoplast solution was loaded on a Hemocytometer, and five 1 mm^2^ squares were observed under a light microscope seven or 14 days after isolation, with three biological replicates. For the callus and shoot regeneration tests, a single treatment consisted of 50 calli with three biological replicates. Results were recorded at different time points depending on experiment, and the detailed information is presented in each corresponding table in the result section. The data were analyzed with ANOVA and Tukey’s test using Minitab (LLC) version 19.2020.1.

### Protoplast Transfection and GFP Detection

To optimize transfection efficiency for field cress, protoplasts were transfected with a vector harboring a gene encoding for green fluorescent protein (GFP) (pCW498-35S-GFiP-OcsT, 14 743 bp (Wood et al., 2009)).

Approximately 150 000 to 200 000 washed protoplasts were mixed with 20–40 µg vector DNA in a 2 ml Eppendorf tube containing 200 µL freshly prepared MMG solution (0.4 M mannitol, 15 mM MgCl_2_, 4 mM MES). Freshly prepared PEG-calcium solution (25% (w/v) PEG4000, 0.4 M mannitol, 0.1 M CaCl_2_) was carefully added to the tube in a 1:1 ratio, and after 5 min the reaction was stopped by addition of 1.5 ml W5 and gentle mixing. The suspension was subsequently centrifuged at 100 *g* for 3 min, and the supernatant was carefully removed. The protoplasts were then re-suspended in 1 ml of MI medium and transferred to 12-well sterile tissue culture plates, wrapped in aluminum foil, and kept in the growth chamber. After 48 h, the protoplasts were observed with a Zeiss LSM 880 Airyscan confocal laser scanning microscope using an EC-Plan-Neofluar 10x/0.30 M27 objective for estimating transfection efficiency. Excitation wavelength was set to 488 nm and detection wavelength was set to 490–585 nm. To ensure that no auto-fluorescence could be observed, non-transfected protoplasts were used as a control. The transfected protoplasts were cultured on the culture media for shoot induction using the optimized regeneration protocol as described above to further verify the protocol.

## Results

### Effect of Basal Medium on Protoplast Regeneration

**FIGURE 1:**
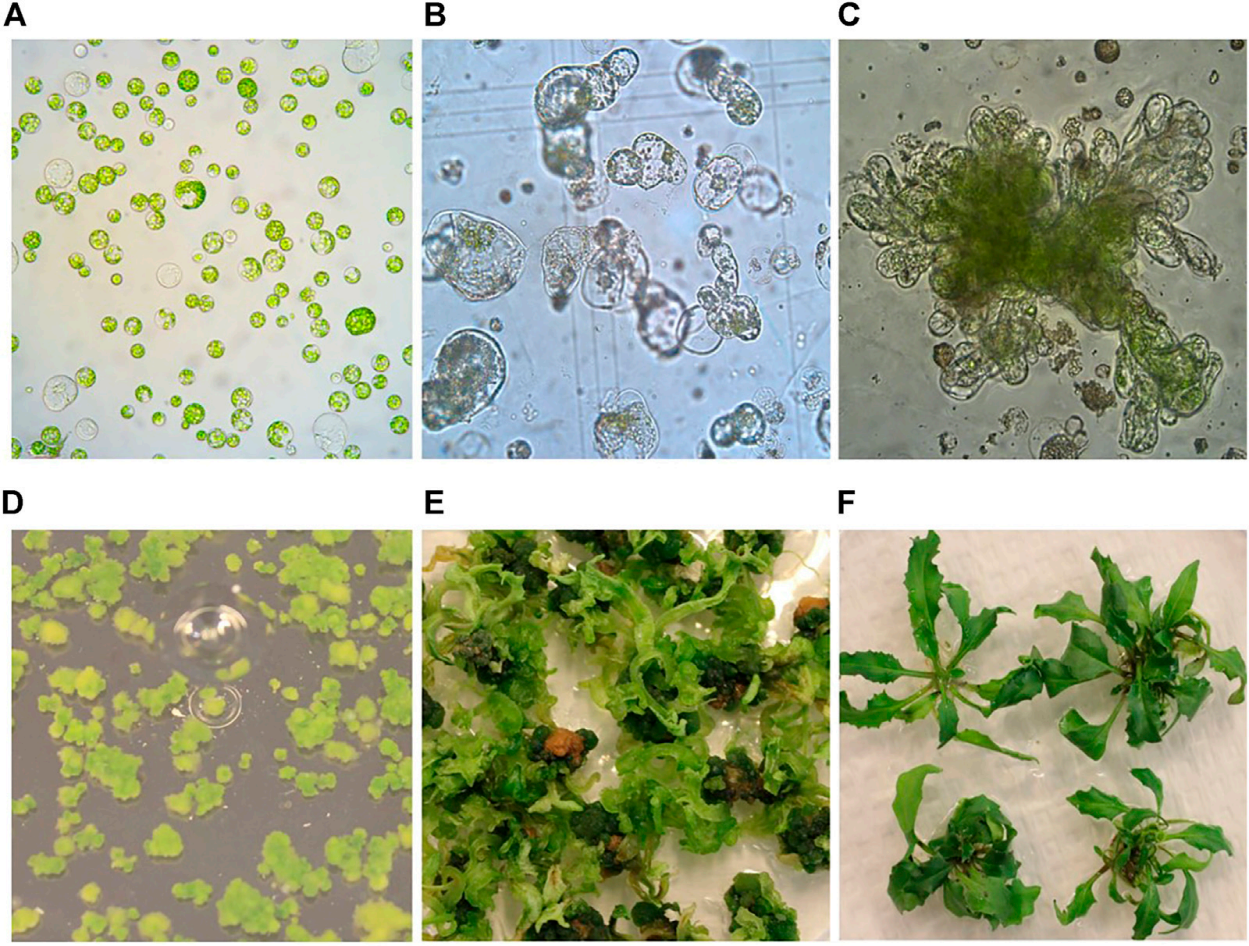
Protoplast isolation, callus formation, and shoot regeneration of field cress. **(A)** Freshly isolated protoplasts (1 day). **(B)** Protoplasts undergoing cell divisions and multiplication (0.5 month). **(C)** Protoplast callus formation (1 month). **(D)** Protoplast colonies (2 months). **(E)** Shoot regeneration from protoplast colonies (4 months). **(F)** Regenerated shoots grown on rooting medium (6 months).

In this study, we have tested widely used media in plant tissue culture, including Murashige and Skoog (MS) (Murashige and Skoog, 1962), Kao (Kao and Michayluk, 1975), B5 (Gamborg et al., 1968) and Nitsch (Nitsch and Nitsch, 1969) for their effects on protoplast regeneration of field cress. The results showed that during the early stage of protoplast culture, Nitsch medium gave the best result in maintaining a higher percentage of viable protoplasts, followed by Kao and B5, while the protoplasts grown in the MS medium become shrunken and the color faded. The protoplast viability was judged by protoplast appearance under a light microscope, which remained green in color and more round in form (Figure 1A). However, the protoplasts did not grow well after 14 days in the Kao medium, while only about 10% of the protoplasts from the B5 medium seemed to be viable. No shoots could be regenerated from the protoplasts initially grown on either MS, Kao, or B5 medium after 4 months. In the Nitsch medium, approximately 80% of the protoplasts grew well after 14 days, significantly higher than in Kao and B5 medium, and 75% of these protoplasts gave rise to shoots after 4 months (Table 1).

## Effect of Plant Growth Regulators (PGRs) in MI Medium on Protoplast Viability

Protoplasts are vulnerable to culture conditions, especially during the early stage of development. The first step in protoplast culture is cell wall formation, during which PGRs play a crucial role. The results of the PGR tests on protoplast growth in this study showed that 0.5 mg L^−1^ 2,4-D combined with 0.5 mg L^−1^ NAA in MI medium was essential to ensure high protoplast viability during the early culture phase. All the other types of PGRs tested would lead to inviable protoplasts, namely being faded in color and shrunken in form (Table 2).

## Effect of PGRs in MII Medium on Protoplast Growth

After the cell wall had formed, the protoplasts would undergo rapid cell division and callus formation, given appropriate growth conditions (Figures 1B, C). We investigated MII media supplemented with different PGRs to determine the most suitable PGR combinations for callus formation. The results showed that the PGR combination of 1.1 mg L^−1^ TDZ with either 0.05 mg L^−1^ 2,4-D or 0.05 mg L^−1^ NAA resulted in the highest percentage of protoplasts with callus formation (Table 3). This result indicates that a relatively lower concentration of auxin was necessary for protoplast development during this stage for field cress.

## Protoplast Regeneration

Difficulty in protoplast regeneration is the major obstacle for the protoplast method to be used for research and crop improvement for most plant species. In order to obtain a high regeneration frequency for field cress, we have investigated the effects of type and concentration of sugars, PGR combinations, and the culture duration in MI, MII, and SIM media on callus formation and subsequent shoot regeneration (Figures 1D–F). The detailed results are presented below.

### Effect of PGR Combinations in SIM Medium on Shoot Regeneration

PGRs is an important factor affecting *in vitro* shoot regeneration. We have thus tested different types and combinations of PGRs to find the best combination for protoplast regeneration of field cress. The results showed that 1.1 mg L^−1^ TDZ alone resulted in a high regeneration frequency (82%), while the combinations of 1.1 mg L^−1^ TDZ with 0.01 mg L^−1^ NAA and 0.1 mg l^−1^ GA_3_, or 2.0 mg L^−1^ BAP with 0.1 mg L^−1^ NAA resulted in very poor regeneration frequencies (5 and 1%, respectively). All other treatments tested did not result in any regeneration. Apart from the 1.1 mg L^−1^ TDZ treatment, all other treatments resulted in hard and/or yellow calli (Table 4).

### Effect of Carbon Source in SIM Medium on Shoot Regeneration

The results showed sugar type and concentration could significantly affect protoplast regeneration frequency (Table 5). Among all the treatments tested, 15 g L^−1^ sucrose gave the best regeneration frequency (80%), followed by 10 g L^−1^ glucose (67%), while 30 g L^−1^ sucrose and 20 g L^−1^ glucose resulted in relatively lower regeneration frequencies (47 and 45%, respectively), suggesting relatively lower concentrations of sugar were more effective in promoting shoot regeneration.

### Effect of Cytokinin in SIM Medium on Shoot Regeneration

As our results showed that TDZ without any auxin was sufficient and efficient in promoting shoot regeneration, we investigated the effect of different concentrations of TDZ alone on shoot regeneration. The results showed that 1.1 mg L^−1^ TDZ was the best concentration tested for shoot regeneration, while higher or lower TDZ concentrations decreased the shoot regeneration frequency. Furthermore, we found that BAP was not effective for shoot regeneration (Table 6).

### Effect of Culture Duration in MI and MII Media on Shoot Regeneration

In this study, we found that the culture duration in MI and MII media played an important role in protoplast regeneration. The results in Table 7 showed that 3 and 5 days culture durations in MI medium gave the best regeneration results, while a culture duration longer than 10 days would inhibit protoplast growth. The culture duration in MII seemed not to have such a critical influence on regeneration frequency, but shoot regeneration was significantly delayed if the culture duration was too long. Given enough time, most of the calli derived from the cultures with an extended period of time in MII medium would eventually develop shoots, with some delay. Approximately 1 month of culture duration in MII medium resulted in the most rapid growth and highest regeneration frequency.

## Protoplast Transfection Efficiency

**FIGURE 2:**
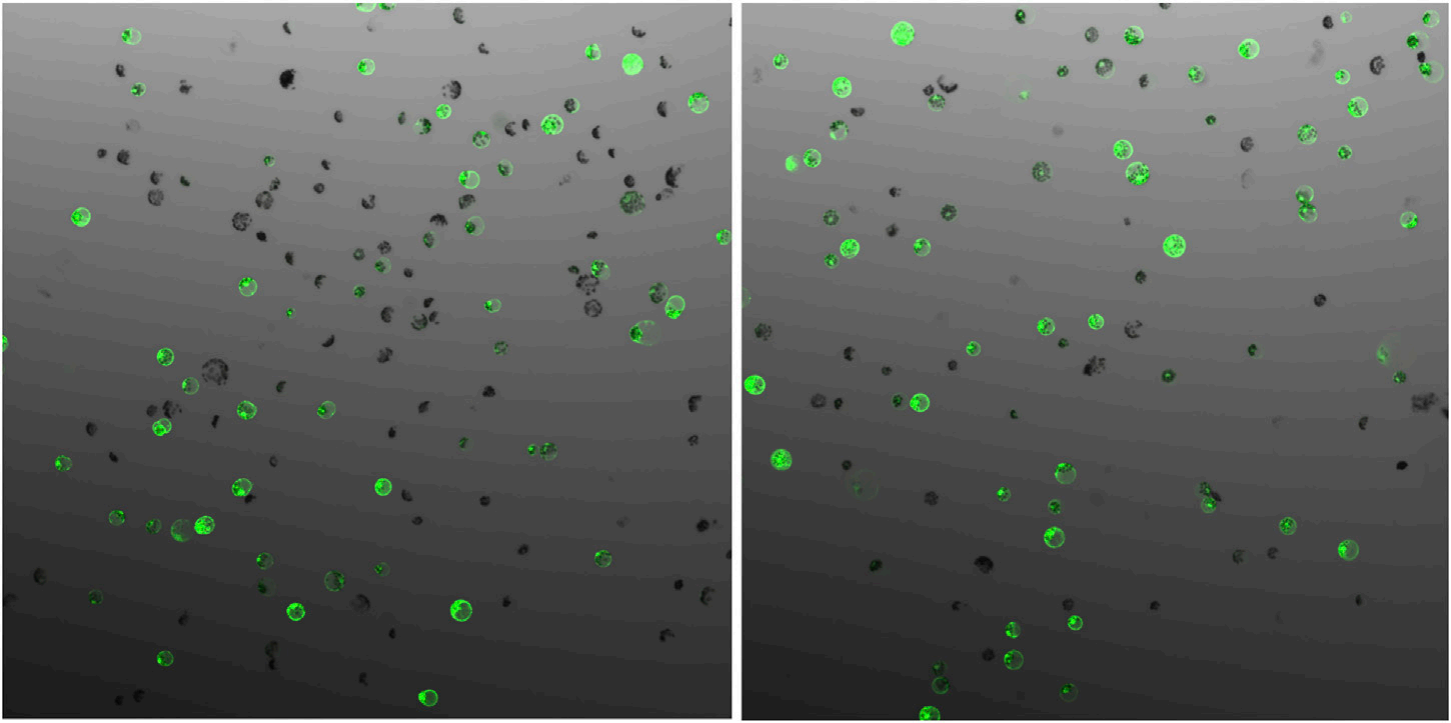
GFP expression 48 h after protoplast transfection of field cress, showing no obvious difference when different vector DNA concentrations (**(A)**, 20 µg and **(B)**, 40 µg) were used for transfection.

**FIGURE 3:**
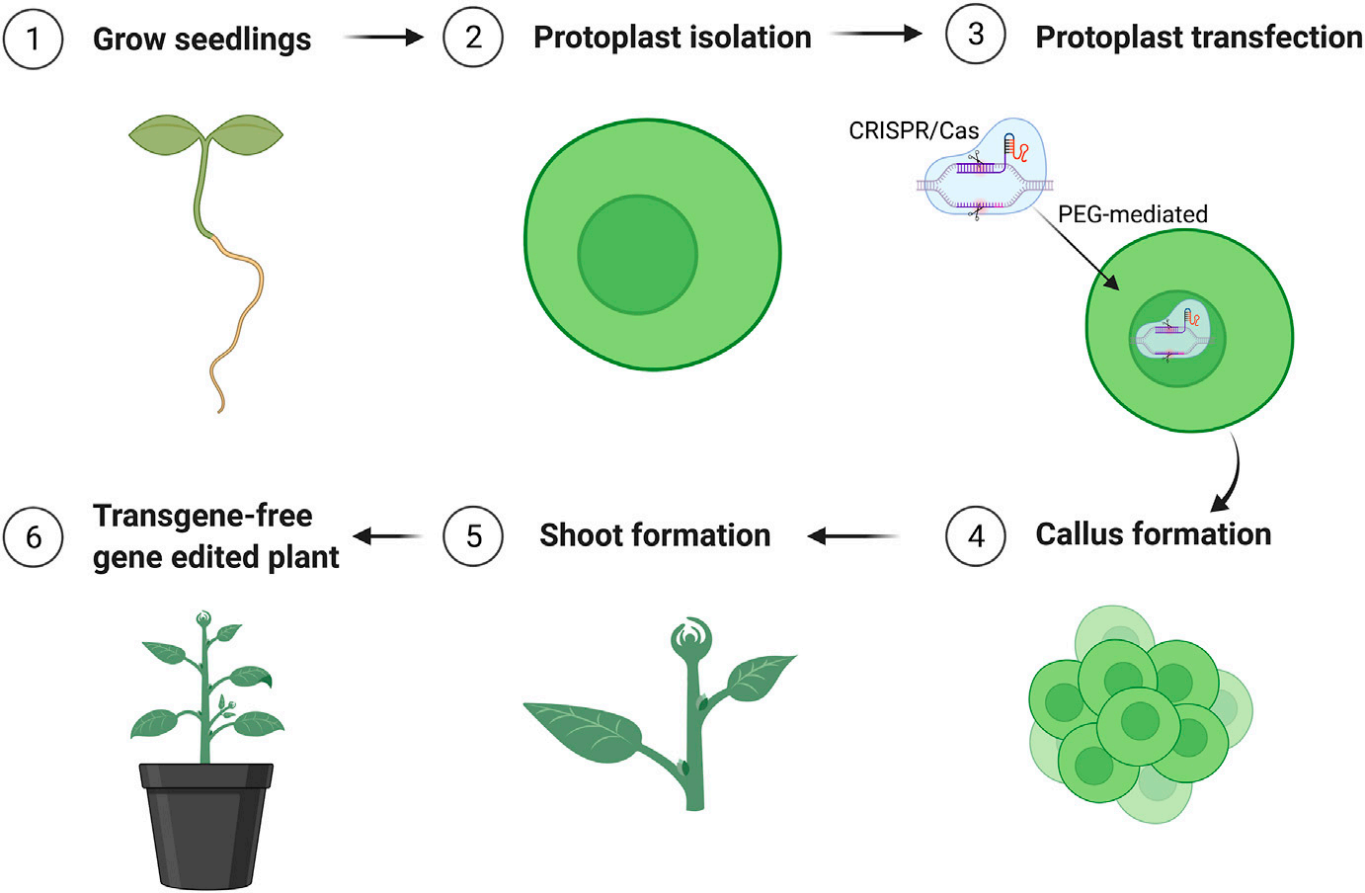
Schematic flowchart of generation of transgene-free mutation lines edited by CRISPR/Cas9 using protoplast approach. The figure was created with BioRender.com.

The parameters affecting transfection efficiency can be species dependent, and it is thus necessary to optimize the transfection protocol for each species. We tested DNA concentrations and PEG/DNA incubation time for field cress in this study. The results showed that a transfection efficiency of 50–80% could be obtained using 25% (w/v) PEG4000, 20–40 µg plasmid DNA, and 5 min incubation time, in which no obvious variation in the GFP protein expression was found between 20 and 40 µg DNA (Figure 2). Furthermore, we were able to use the regeneration protocol described above to regenerate shoots from transfected protoplasts with normal regeneration efficiency. A flowchart from protoplast isolation to generation of edited transgene-free plants is presented in Figure 3 to facilitate readers’ understanding of protoplast-based gene editing. This protocol will be very valuable for our ongoing work on genetic modification of important traits in field cress by CRISPR/Cas9.

## Discussion

The interest in applying protoplast culture technique for plant research and crop improvement has increased alongside the increased application of gene editing by CRISPR/Cas9, as it can generate transgene-free mutant lines. However, due to the major obstacle in protoplast regeneration, application of the protoplast method for gene editing is still very limited for trait improvement for most important crops. It is very challenging to develop an efficient protoplast regeneration protocol for the majority of crop species. Protoplasts from different species, genotypes, and different tissues, may require different culture conditions for successful regeneration. Some of the critical parameters include protoplast isolation method, medium composition, culture duration, and callus development phase suitable for shoot induction, which has been shown to be critical for successful protoplast regeneration in our earlier report on rapeseed (Li et al., 2021). To obtain a high regeneration frequency it is often necessary to optimize the abovementioned important parameters and other culture conditions, which is very time- and labor-intensive. There is so far, to the best of our knowledge, no published report available on protoplast culture for field cress. In this study, we have systemically investigated some important factors affecting protoplast culture and regeneration, and have established a highly efficient and relatively simple protocol for protoplast regeneration and transfection for the species. This protocol would provide a solid foundation for further improvement of this potential novel oilseed crop through gene editing technologies, and also provide important information for developing protoplast regeneration protocols for other plant species.

Protoplasts can be isolated from various tissues and organs of plants, such as leaves, roots, petioles, cotyledons, hypocotyls, embryos, and microspores. The use of some types of tissues, such as roots and hypocotyls, usually requires a large amount of materials to obtain satisfactory protoplast yields, which makes them unpractical to use (Klimaszewska and Keller, 1987; Eeckhaut et al., 2013). Leaf tissues often provide satisfactory protoplast yields, and protoplast isolation from leaves is thus preferred by most researchers (Yoo et al., 2007; Nicolia et al., 2015; Lin et al., 2020; Molla et al., 2020). In our preliminary studies, we tested both leaves and hypocotyls for protoplast isolation and culture for field cress. The protoplasts from hypocotyls could be isolated and developed into protoplast colonies, but it required several fold more plant materials to yield a satisfactory quantity of protoplasts compared with leaves. We thus only used leaves for protoplast isolation in further studies. Although not tested systematically, it seemed that fully opened true leaves from 3–4 week old seedlings was the most suitable material for protoplast isolation and subsequent regeneration. Leaves from more than 4-week old seedlings could also be used successfully, but the regeneration frequency might be compromised.

Protoplasts are naked cells without cell wall, which are very vulnerable to certain culture conditions. The first step in protoplast culture is to promote cell wall formation, and then rapid cell division and callus formation. The cell wall formation starts within a few hours after protoplast isolation and it may take several days to complete the process (Zaban et al., 2013). Protoplast cell necrosis usually occurs during this period if culture conditions are unfavorable. We found that both the MI medium composition and the culture duration in the medium are crucial for protoplast viability, growth, and subsequent regeneration. It has been reported that for successful culture of *Brassica* protoplasts, both 2,4-D and NAA are necessary at the early culture stages to sustain protoplast survival and induce cell division, and that the appropriate ratio of NAA to 2,4-D is genotype-dependent (Glimelius, 1984). In this study, our results showed that equal amounts of 2,4-D and NAA (0.5 mg L^−1^ of each) was the most suitable for maintaining protoplast viability and subsequent regeneration of field cress. Moreover, the culture duration in MI medium also appears to be crucial for successful protoplast regeneration. In this study, 3–5 days gave the best results, as longer culture duration would cause the cells to stop growing. The culture duration in MII appeared not to be so critical compared to MI for protoplast regeneration in field cress, as a prolonged culture duration would mainly delay shoot regeneration.

Low regeneration frequency is the main obstacle affecting application of the protoplast approach in research and trait improvement for most economically important crops. Under suitable culture conditions, protoplasts would undergo a series of differentiation stages and finally form shoots. Among the factors affecting protoplast regeneration, PGRs are of critical importance. Although a high cytokinin/auxin ratio is required for shoot regeneration, this ratio often varies from genotype to genotype (Kao and Seguin-Swartz, 1987), apparently due to differencen in concentrations of both endogenous hormones. In this study, we found that 1.1 mg L^−1^ TDZ alone in the SIM medium was sufficient to give the highest regeneration frequency among all treatments tested. This is in agreement with our previous study, in which 1.1 mg L^−1^ TDZ without auxin resulted in a high regeneration frequency when hypocotyls were used as explants for genetic transformation (Ivarson et al., 2013). For sugar tests, we observed that sucrose gave a significantly higher regeneration frequency than glucose. Sucrose is often efficiently used in most of the crop species in tissue culture, likely because it is the most common carbohydrate synthesized and transported in the phloem sap of many plants. In case of the protoplasts, it is also likely that sucrose may better facilitate growth and development due to its impact on cell osmolarity (Yaseen et al., 2013).

The density of protoplasts in MI and MII media appears to be an important factor affecting the protoplast viability. Some studies suggested that relatively high protoplast culture densities would promote cell growth and division (Chuong et al., 1985; Kiełkowska and Adamus, 2012). It could be that growing protoplasts stimulate growth and mitotic division of adjacent cells by releasing growth factors into the medium (Davey et al., 2005). In this study, we also observed that a low protoplast density would usually result in poor protoplast viability during the early stage of cultures. However, a too high protoplast density would results in brownish protoplast colonies. This is probably due to competition for limited available nutrients in the medium, resulting in a large number of protoplasts failing to undergo cell divisions. In the case of field cress, we found in this study that the suitable protoplast plating density was 0.4–0.6 million protoplasts per ml. When performing transfections it was necessary to increase the initial protoplast density to 0.75–1.0 million per ml for better regeneration, as the PEG-incubation would result in a loss of protoplasts.

In conclusion, through optimizing various important culture conditions, we have developed a highly efficient protoplast regeneration and transfection protocol for field cress. This protocol will provide a solid foundation for using the protoplast approach for molecular studies and developing CRISPR/Cas9-edited transgene-free mutant lines of field cress. The protocol would also be helpful in establishing protoplast regeneration protocols for other related plant species.

## Data Availability

The raw data supporting the conclusions of this article will be made available by the authors, without undue reservation.
