# Corrigendum: Establishment of an efficient protoplast regeneration and transfection protocol for field cress (*Lepidium campestre*)

**DOI:** 10.3389/fgeed.2023.1183791

**Published:** 2023-03-27

**Authors:** Sjur Sandgrind, Xueyuan Li, Emelie Ivarson, Annelie Ahlman, Li-Hua Zhu

**Affiliations:** Department of Plant Breeding, Swedish University of Agricultural Sciences, Lomma, Sweden

**Keywords:** Braccicaceae, CRISPR/Cas9, oilseed crop, domestication, protoplast regeneration, transfection

Incorrect Funding

In the published article, there was an error in the **Funding** statement. The original funding statement missed the parentheses for the full name of FORMAS and grant number, “FORMAS (The Swedish Research Council for Sustainable Development) grant 2016-01401.”

The correct **Funding** statement appears below

“Financial support to this research by MISTRA (The Foundation for Strategic Environmental Research) and The Swedish University of Agricultural Sciences through Mistra Biotech program, TC4F (Trees and Crops for the Future), a strategi research area at SLU, supported by the Swedish Government, FORMAS (The Swedish Research Council for Sustainable Development) grant 2016-01401 and Einar and Inga Nilsson’s foundation as well as The Royal Physiographic Society of Lund is highly acknowledged.”

The authors apologize for this error and state that this does not change the scientific conclusions of the article in any way. The original article has been updated.

